# Extraosseous Ewing's Sarcoma of the Chest Wall: A Case Report and Review of Literature

**DOI:** 10.7759/cureus.95239

**Published:** 2025-10-23

**Authors:** Yan Feng, Yan Liang, Rui Wang, Zhiren Chen

**Affiliations:** 1 Radiology, Jilin Province People's Hospital, Changchun, CHN; 2 Nuclear Medicine, Changchun Cancer Hospital, Changchun, CHN; 3 Diagnostic Imaging and Nuclear Medicine, Changchun Cancer Hospital, Changchun, CHN

**Keywords:** chest wall, diagnosis, ewing’s sarcoma, extraosseous, mri

## Abstract

This case report presents a 15-year-old boy with extraosseous Ewing's sarcoma who initially experienced right chest wall pain and fever. Imaging revealed a soft-tissue mass with ring-like enhancement and mild rib destruction. Thoracoscopic resection was performed, and paraffin pathology confirmed the diagnosis of extraosseous Ewing's sarcoma. The patient had a smooth postoperative recovery. This case highlights the diagnostic challenges and therapeutic strategies associated with Ewing's sarcoma and emphasizes the importance of early and accurate diagnosis to improve clinical outcomes.

## Introduction

Ewing's sarcoma (ES) is a rare and highly malignant small round cell tumor that primarily affects children and adolescents [1]. While it most commonly arises in the long bones and pelvis, it can also develop in extraosseous soft tissues, where it is referred to as extraosseous Ewing's sarcoma (EES). Although EES accounts for only 20%-30% of all ES cases and represents less than 1% of all sarcomas, the chest wall is recognized as one of its more frequently encountered anatomical sites [2,3]. Due to its nonspecific clinical presentation and imaging features that often mimic inflammatory lesions or other malignancies, it is frequently misdiagnosed. This diagnostic challenge is compounded by the tumor's diverse manifestations across different locations, as highlighted in recent literature [4]. This study explores the diagnosis and treatment of EES of the chest wall with reference to relevant literature.

## Case presentation

The patient, a 15-year-old male adolescent, presented with unexplained chest pain for 5 days and fever for 3 days, with a peak temperature of 39°C. Laboratory tests revealed a slightly elevated white blood cell count (15×10⁹/L) and significantly elevated C-reactive protein (143.1 mg/L) (Table 1).

**Table 1:** Laboratory findings of a 15-year-old male patient with corresponding reference ranges.

Test	Result	Normal Range	Interpretation
Body Temperature	39.0 °C	36.5 – 37.5 °C	Fever
White Blood Cell Count	15 × 10⁹/L	4.0 – 10 × 10⁹/L	High (Leukocytosis)
C-Reactive Protein (CRP)	143.1 mg/L	< 5 mg/L	Markedly High

**Figure 1:**
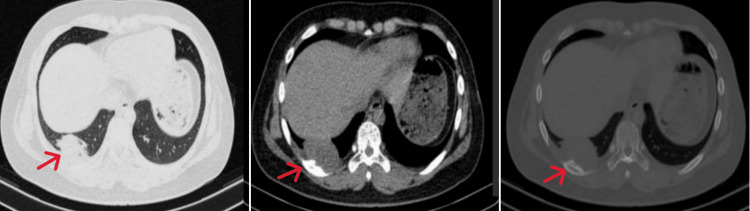
Extraosseous Ewing's sarcoma (EES) chest CT scan of the right chest wall. A. Axial lung window. B. Axial mediastinal window. (A-B) Soft tissue mass is seen in the right lower lung and the posterolateral base of the chest wall. C. Axial bone window. Mild bone destruction is seen near the right ninth rib.

No abnormalities were detected in immunoglobulin M(IgM) antibodies for *Mycoplasma pneumoniae *and Chlamydia, tuberculosis antibodies, tumor markers, coagulation profile, routine immunological parameters, liver and kidney function tests, ion levels, or urinalysis. Imaging examination: chest computed tomography(CT) revealed a soft-tissue density mass in the right lower lung base, posterior and lateral chest wall. The lesion appeared similar in size on mediastinal and lung window images (Figures 1A-1B), while the bone window showed mild bone destruction of the right ninth rib (Figure 1C).

**Figure 2:**
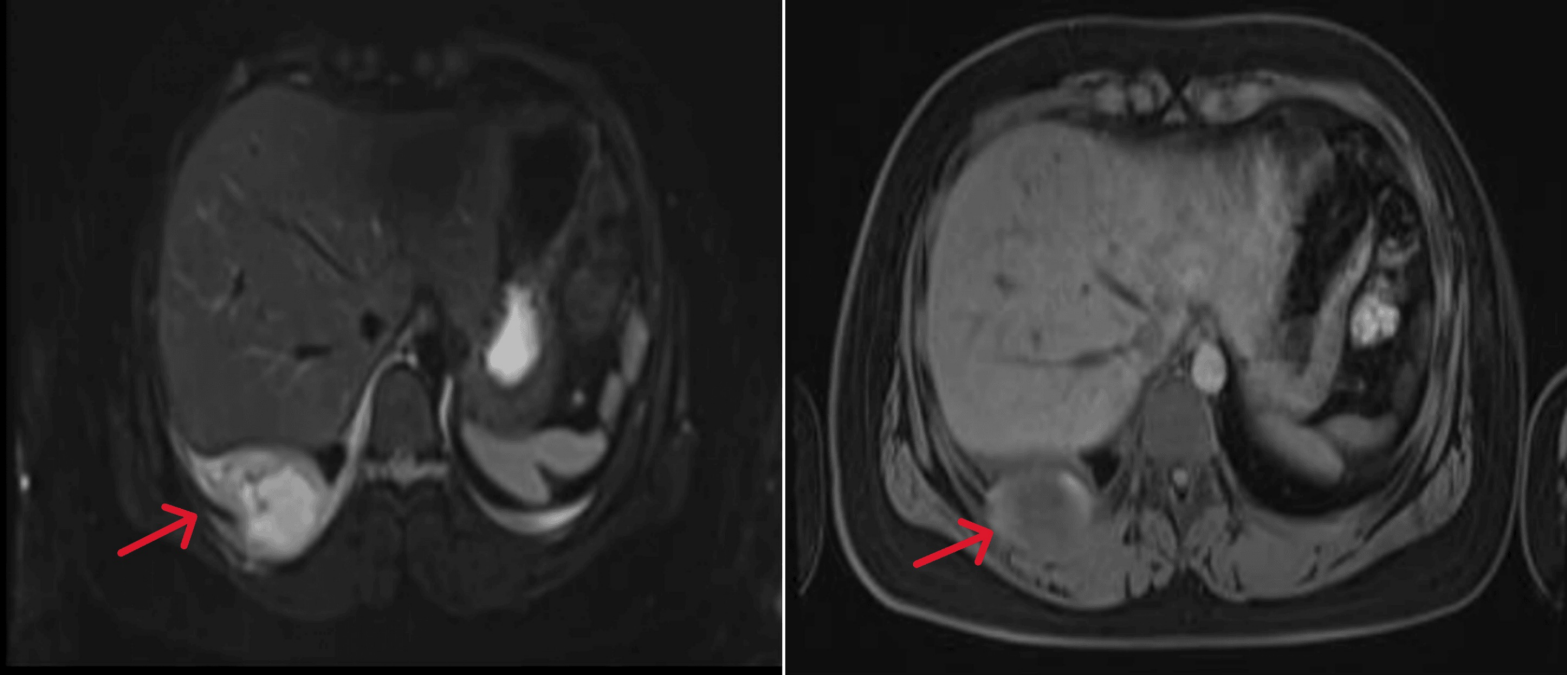
Extraosseous Ewing's sarcoma (EES) magnetic resonance imaging (MRI) of the right chest wall non-enhanced examination. A. Axial T2-weighted imaging fat suppression (T2WI-FS) showed high and slightly low signal; B. Axial T1-weighted imaging (T1WI) showed slightly low signal.

**Figure 3:**
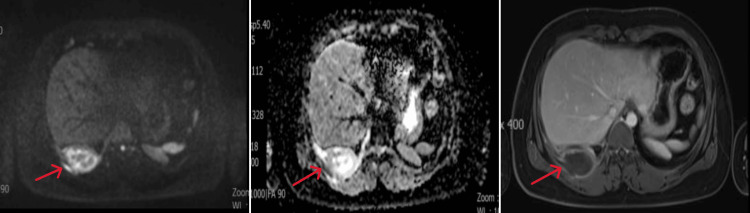
Extraosseous Ewing's sarcoma (EES) magnetic resonance imaging (MRI) of the right chest wall enhanced and non-enhanced examination. A. Axial diffusion weighted imaging (DWI) showed mixed edge high signal; B. Axial apparent diffusion coefficient (ADC) showed edge low signal; C. Axial vT1-weighted imaging (T1WI)-enhanced scan showed edge enhancement, but no enhancement was seen in the central necrotic area.

Non-contrast and contrast-enhanced magnetic resonance imaging (MRI) of the mediastinum showed that the right lower chest wall mass showed high signal on T2-weighted imaging fat suppression (T2WI-FS) images (Figure 2A), slightly low signal on T1-weighted imaging (T1WI) (Figure 2B), mixed edge high signal on diffusion weighted imaging (DWI )(Figure 3A), and low signal on apparent diffusion coefficient (ADC) edge (Figure 3B). Enhanced imaging (Figure 3C) showed peripheral enhancement of the lesion with central necrosis and involvement of the adjacent pleura.

The maximum cross-sectional size of the mass measured approximately 5.3 cm × 3.5 cm. A small right-sided pleural effusion was also noted. Based on these findings, a tumor in the right chest wall was suspected. A positron emission tomography/computed tomography (PET/CT) scan obtained at an external institution demonstrated hypermetabolism in the patient's right chest wall and adjacent ninth rib, strongly suggesting malignancy. The patient underwent thoracoscopic resection of the right parietal pleural tumor and a wedge resection of the right lower lung lobe under general anesthesia.

**Figure 4:**
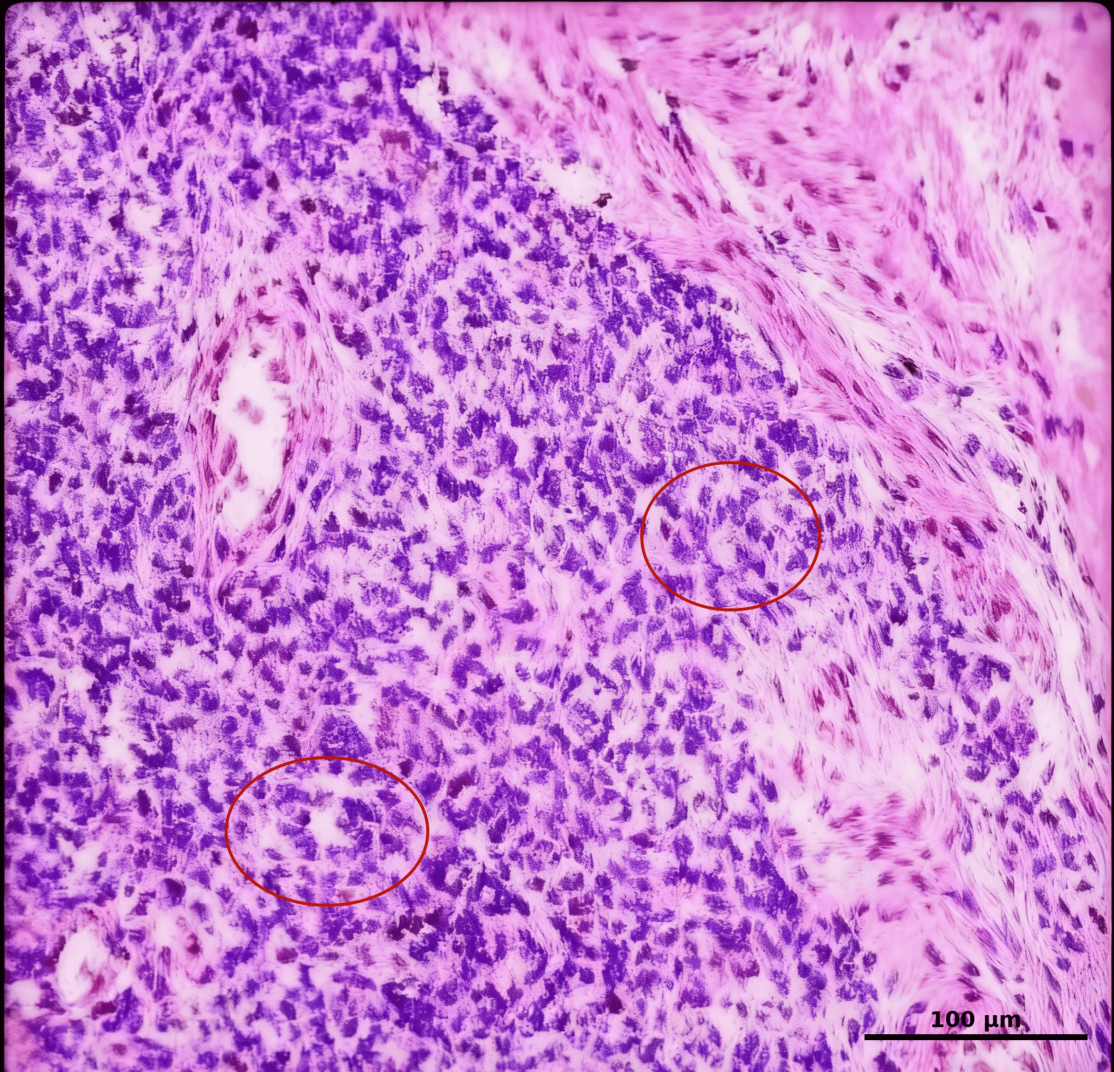
Pathology H&E, ×400 (10×40). The tumor cells exhibit a diffuse infiltrative growth pattern. The neoplastic cells are round or ovoid in shape with scant cytoplasm, and some cells show clear cytoplasm. Fibrous septa are observed. Tumor cells are arranged around blood vessels, forming Homer-Wright rosette structures (red circle).

Rapid pathology showed malignant small-cell tumor lesions composed mostly of necrotic tissue. Paraffin pathology (Figure 4) showed that the right chest wall tumor, combined with morphology and immunohistochemistry (IHC) results, was consistent with EES, with a large amount of tumor necrosis, and the tumor had invaded the right lower lobe lung tissue with adjacent pleural thickening. IHC: Vimentin (Vim, +), CD99 (+), Synaptophysin (Syn, weak +), Bcl-2 (weak +), WT-1 (weak +), Ki-67 (hot spot area ≈70%), Cytokeratin (CK, -), Epithelial Membrane Antigen (EMA, -), Neuron-Specific Enolase (NSE, -), Desmin (-), Smooth Muscle Actin (SMA, -), Leukocyte Common Antigen (LCA, -), Myeloperoxidase (MPO, -), Terminal deoxynucleotidyl transferase (TdT, -).

## Discussion

Ewing's sarcoma (ES) is a high-grade malignant small round cell tumor with an extremely low incidence, accounting for less than 1% of all sarcomas [1]. It can be categorized into Ewing’s sarcoma of bone (ESB), EES, malignant small cell tumor of the chest wall (Askin tumor), and primitive neurodermal tumor (PNET) based on soft tissue. EES is a relatively rare malignant small round cell tumor that arises in deep soft tissues and accounts for approximately 20%-30% of all ES cases. It shares similar microscopic morphology, ultrastructure, and chromosomal abnormalities with ESB and is considered a unique manifestation of the latter [3]. EES typically occurs in deep soft tissues such as the retroperitoneum, cervical spine, paraspinal region, chest, and lower limbs. It is characterized by rapid growth, aggressive metastasis, and a poor prognosis due to its high invasiveness. Most cases occur in children, adolescents, and young adults, with over 90% of patients being between the ages of 4 and 25.

The clinical presentation of pulmonary EES varies widely and may include symptoms such as dyspnea (54.3%), cough (55.3%), chest pain (50%), fever (17%), and hemoptysis (14.9%) [4]. Symptoms of metastatic disease, including general malaise, weakness, fever, anemia, and weight loss [5], are nonspecific and often indistinguishable from those of other malignancies. This nonspecific symptomatology contributes to the frequent misdiagnosis of EES in clinical practice.

Imaging examinations play a crucial role in the diagnosis, staging, treatment monitoring, and efficacy evaluation of EES. CT scans typically reveal uniform or heterogeneous iso- or hypodense masses. The heterogeneous density may be attributed to cystic changes or necrosis, while calcification is uncommon, observed in only 10% of previously reported cases [6]. Following contrast administration, the mass usually demonstrates heterogeneous mild to moderate progressive enhancement, with no enhancement noted in areas of cystic necrosis. Multiplanar reconstruction (MPR) using multislice spiral CT is helpful for accurately locating the mass. MRI is the preferred imaging modality for EES, as it provides detailed information on the lesion's location, size, margins, and internal signal characteristics. On T1WI, the lesion typically appears isointense or slightly hypointense relative to muscle. T2WI generally shows a slightly hyperintense signal, while T2WI-FS reveals a markedly hyperintense signal.

Enhanced imaging demonstrates uniform or heterogeneous enhancement of the lesion. Due to its highly malignant nature, the tumor can invade adjacent thoracic structures and pulmonary parenchyma, leading to lung collapse or atelectasis, and may also cause destruction of nearby vertebrae and ribs. EES primarily metastasizes via lymphatic pathways, with hematogenous spread being rare [7-9]. Fluorodeoxyglucose positron emission tomography (FDG PET) demonstrates increased radionuclide uptake and is useful for detecting primary lesions, as well as for identifying residual or recurrent tumors after surgery and metastatic lesions. Histologically, the tumor was composed of small, round, blue cells. Immunohistochemical (IHC) analysis is crucial for diagnosis. The tumor cells demonstrated strong positivity for CD99 (also known as MIC-2), a cell surface glycoprotein that typically exhibits a characteristic membranous pattern, and for vimentin, a cytoskeletal protein.

Nuclear expression of FLI-1, a transcription factor from the Ets family that is frequently involved in the pathogenesis of Ewing sarcoma, was also observed. These markers serve as relatively specific diagnostic markers for ES. In contrast, the tumor cells were negative for S-100 protein, neurofilament protein, and UEA-1, which aids in excluding other differential diagnoses such as melanoma or neurogenic tumors. In some cases, neuro-specific enolase may be expressed [9]. The definitive diagnosis often relies on molecular confirmation of the characteristic translocation, such as t(11;22)(q24;q12), combined with the typical IHC profile, to distinguish EES from other small round blue cell tumors [10]. In this case, the positivity for CD99 and vimentin was consistent with a diagnosis of EES; however, genetic testing for the specific translocation was not performed for this patient.

In this case, the solitary mass on the right chest wall showed uneven density and signal characteristics, with irregular enhancement, areas of necrosis, significant peripheral enhancement, mild destruction of the adjacent rib, and invasion of the lung parenchyma. While the imaging features of EES lack specificity, both CT and MRI are valuable for locating the tumor, analyzing its internal characteristics, determining its extent, detecting invasion of adjacent structures or metastasis to distant organs, and assessing the feasibility of surgical intervention. A comprehensive approach combining clinical and imaging findings, along with IHC staining and cytogenetic analysis, is essential for confirming the diagnosis, guiding treatment decisions, improving prognosis, and increasing the survival rate.

EES must be differentiated from other soft tissue malignancies. Neuroblastoma, the most common extracranial solid tumor in children, accounts for approximately 6%-8% of all childhood tumors and 15% of childhood cancer-related deaths. The majority of cases (90%) are diagnosed before the age of 5 years [11,12]. On CT, the density of the mass is uneven, with spotty, nodular, or localized calcifications. MRI reveals mixed signals, with T1WI predominantly showing low signal and T2WI showing high signal. Focal necrosis areas are visible, with mild to moderate enhancement, and the mass may directly invade the chest wall, extending to the adjacent neural foramen [13].

Rhabdomyosarcoma, the most common soft tissue sarcoma in children and adolescents, is less common in adults. These masses typically arise in the muscle belly, growing infiltratively along muscle fibers towards their ends. On CT, they appear as irregular, iso- or low-density soft tissue masses with unclear borders. The masses may be cystic or necrotic, often growing invasively and causing surrounding bone destruction. After contrast administration, the masses exhibit mild to moderate heterogeneous enhancement, with tortuous vascular shadows seen during the arterial phase. The mass may also display patchy low-density, non-enhanced areas. On MRI, T1WI shows equal or low signal, while T2WI shows high signal, with uniform or inhomogeneous signals. DWI demonstrates restricted diffusion, and the enhanced mass shows mild to moderate heterogeneous enhancement [14].

Malignant lymphoma, typically seen in elderly patients, presents with extensive bone destruction, reactive sclerosis, and local periosteal reaction, accompanied by large soft tissue masses, often surpassing the extent of bone destruction. CT, MRI, and PET/CT imaging will remain essential tools for the diagnosis, staging, treatment response monitoring, and surveillance of these malignant tumors.

The primary treatment approach is multidisciplinary, beginning with a biopsy to confirm the diagnosis, followed by neoadjuvant chemotherapy aimed at reducing the size of the primary lesion and eliminating micrometastases. Surgery is the cornerstone of local control, but decisions are influenced by various factors, including the tumor stage, the patient's age, tumor size, location, and involvement of adjacent vital structures. Whether the tumor involves neurovascular bundles and whether the primary lesion can be completely resected with clear margins are also important considerations.

In addition to surgery and neoadjuvant chemotherapy, adjuvant treatment plays a vital role in improving long-term outcomes. The standard management of Ewing’s sarcoma includes systemic multi-agent chemotherapy combined with local control measures such as surgery and/or radiotherapy. Common regimens include vincristine, doxorubicin, and cyclophosphamide alternating with ifosfamide and etoposide (VDC/IE). Adjuvant radiotherapy is recommended when surgical margins are positive, resection is incomplete, or postoperative imaging suggests residual disease. Postoperative CT or MRI helps assess residual lesions and guides further management. A multidisciplinary team approach is crucial for optimizing treatment and improving survival.

## Conclusions

Although primary EES originating from the chest wall is rare, the final diagnosis must be confirmed through tissue morphology, IHC, and molecular genetic analysis. Imaging plays an irreplaceable role in determining the size, composition, relationship with adjacent structures, and blood supply of the lesion, as well as in evaluating treatment efficacy and follow-up. This case underscores the importance of maintaining a high level of suspicion for ES when encountering unexplained soft tissue masses in children and adolescents, particularly when conventional treatments are ineffective. Imaging specialists and clinicians should enhance their ability to recognize rare malignant tumors and, when necessary, perform a pathological biopsy to confirm the diagnosis. This approach increases diagnostic accuracy and enables the timely selection of the most appropriate treatment strategy, ultimately improving patient outcomes.

Given the generally poor prognosis of Ewing sarcoma, early diagnosis, standardized multidisciplinary treatment, and long-term close follow-up are crucial for improving patient outcomes. Regular postoperative imaging, including MRI or CT scans of the primary site and chest imaging every 3-6 months during the first two years and annually thereafter, facilitates timely detection of local recurrence or distant metastasis. Early identification of relapse allows prompt initiation of salvage therapy and may improve overall survival.
